# Intratumoral treatment of one tumor lesion with LTX-315 induces complete tumor regression and long-term specific protective immune responses in a metastatic rodent tumor model

**DOI:** 10.1186/2051-1426-2-S3-P236

**Published:** 2014-11-06

**Authors:** Öystein Rekdal, Janne Nestvold, Meng Yu Wang, Ketil A Camilio, Baldur Sveinbjörnsson, Gunnar Kvalheim

**Affiliations:** 1 Lytixbiopharma, Oslo, Norway, Oslo, Norway; 2 Dept. of Anatomy, Inst. Basal Medical Sciences, University of Oslo, Oslo, Norway; 3 Dept of Cellular Therapy, Inst. Cancer Research, The Norwegian Radium Hospital, Oslo, Norway; 4 Molecular Inflamamtion Research Group, Inst. Medical Biology, University of Tromsö, Tromsö, Norway

## 

Host defense peptides are naturally occurring peptides that have an important function in innate immune responses in almost every life form. Recently it has been documented that several host defense peptides have anticancer activity. Based on a naturally occurring host defense peptide, we have *do novo *designed the short chemically modified peptide LTX-315. We have demonstrated that LTX-315 induces an immunogenic type of cell death with subsequent release of danger signals (e.g. HMBG1, ATP and Cytochrome C) and tumor associated antigens (TAA's). In addition LTX-315 also has the ability to directly modulate immune-responses.

In a novel rat mescenhymal sarcoma model (rTMSC) we demonstrate that LTX-315 induces a complete tumor regression by intratumoral (i.t.) injection. Studies on treated tumor tissue confirmed massive necrosis and infiltration of immune cells. Successfully treated animals were protected against re-challenge with the tumor cell type treated, but not against other types of tumor cells. Moreover, tumor resistance could be adoptively transferred by spleen cells from LTX-315-treated animals. The resistance was abrogated by depletion of T-lymphocytes.

To clarify whether intratumoral injection of LTX-315 in one tumor lesion can have an effect on metastatic disease, intraperitoneal tumor and two subcutaneous tumors were established in the animals. Thereafter, LTX-315 was injected into one of the subcutaneous lesion and tumor growth assessed by living imaging. The results showed that LTX-315 eradicated all three lesions and the animal went into durable complete remission.

We propose that by targeting tumor locally LTX-315 can be used for individualized therapeutic *in situ *vaccination against cancer.

